# Impaired glymphatic clearance measured from DTI‐ALPS in neurodegenerative and cerebrovascular disease

**DOI:** 10.1002/alz70856_104492

**Published:** 2025-12-26

**Authors:** Daniela Andriuta, Joel Ramirez, Lauren Abby Woods, Min Su Kang, Stephanie Berberian, Fuqiang Gao, Christopher JM Scott, Dana N Broberg, Robert Bartha, Richard H. Swartz, Mario Masellis, Sandra E. Black, ONDRI Investigators

**Affiliations:** ^1^ Department of Neurology, Amiens University Medical Center, Amiens, France; ^2^ Laboratoire de Neurosciences Fonctionnelles et Pathologies (UR UPJV 4559), Jules Verne University of Picardy, Amiens, France; ^3^ Dr. Sandra E. Black Centre for Brain Resilience and Recovery, Toronto, ON, Canada; ^4^ Dr. Sandra Black Centre for Brain Resilience & Recovery, Sunnybrook Research Institute, Toronto, ON, Canada; ^5^ Western University, London, ON, Canada; ^6^ Robarts Research Institute, London, ON, Canada; ^7^ Division of Neurology, Department of Medicine, Sunnybrook Health Sciences Centre, Toronto, ON, Canada; ^8^ Dr. Sandra Black Centre for Brain Resilience & Recovery, Toronto, ON, Canada

## Abstract

**Background:**

Impaired glymphatic clearance has been associated with neurodegenerative diseases causing dementia. The diffusion tensor image analysis along the perivascular space (DTI‐ALPS) index has been proposed as a non‐invasive measure to assess glymphatic function. This preliminary analysis aimed to examine correlations with DTI‐ALPS and changes in brain atrophy in the Ontario Neurodegenerative Disease Research Initiative (ONDRI) cohort.

**Methods:**

The ONDRI study participants (*n* = 125; age = 54.9+/‐7.5 years; 76% male) in this analysis included clinically diagnosed patients with Alzheimer's disease/mild cognitive impairment (ADMCI; *n* = 44), cerebrovascular disease (CVD; *n* = 68), and frontotemporal dementia (FTD; *n* = 13). DTI images were preprocessed with the FSL Diffusion Toolbox, and the ALPS indices (mean, left and right) were calculated using diffusion data from the projection and association fiber regions. Lower DTI‐ALPS indices having been linked to reduced glymphatic function. Fractional perivascular space (PVS) and white matter hyperintensity (WMH) volumes were extracted from MRI. Whole brain atrophy was assessed using the brain parenchymal fraction (BPF), including one‐year change in BPF. ANOVA, Pearson correlation, and linear regression models were used to analyze the data. Demographics (age, sex, education, handedness) and vascular risk factors (hypertension, diabetes, hypercholesterolemia, smoking, waist‐to‐hip ratio) were also collected.

**Results:**

Mean DTI‐ALPS was highest in ADMCI and lowest in CVD (ADMCI=1.49; FTD=1.44, CVD=1.37; contrasts: ADMCI vs. CVD, *p* <0.05, others n.s.). At baseline, partial correlations controlling for demographics, clinical diagnosis, and vascular risk factors showed mean DTI‐ALPS was significantly correlated with baseline BPF (r=.204, *p* = 0.028) and baseline WMH (r=‐0.287, *p* = 0.002), but not with baseline PVS. Baseline right DTI‐ALPS was significantly associated with delta BPF (β=0.209, *p* = 0.019), independent of demographics, clinical diagnosis, vascular risk factors, and baseline WMH.

**Conclusion:**

As a possible measure of glymphatic clearance functioning along the perivascular space, our preliminary results showed that baseline DTI‐ALPS measures were associated with one‐year change in whole brain atrophy in patients with neurodegenerative and cerebrovascular disease. This finding was independent of demographics, clinical diagnosis, and vascular risk factors. Future planned analysis of the ONDRI cohort will examine DTI‐ALPS associations with cognitive decline in the presence of white matter changes and elevated plasma glial fibrillary acidic protein as an inflammation marker.

